# High plasmid variability, and the presence of IncFIB, IncQ, IncA/C, IncHI1B, and IncL/M in clinical isolates of *Klebsiella pneumoniae* with *bla*_KPC_ and *bla*_NDM_ from patients at a public hospital in Brazil.

**DOI:** 10.1590/0037-8682-0397-2020

**Published:** 2020-10-21

**Authors:** Érica Maria de Oliveira, Elizabeth Maria Bispo Beltrão, Alexsandra Maria Lima Scavuzzi, Josineide Ferreira Barros, Ana Catarina Souza Lopes

**Affiliations:** 1 Universidade Federal de Pernambuco, Centro de Ciências Médicas, Área de Medicina Tropical, Recife, PE, Brasil.; 2 Hospital Agamenon Magalhães, Recife, PE, Brasil.

**Keywords:** Klebsiella pneumoniae, Antimicrobial resistance, Plasmids, Incompatibility groups

## Abstract

**INTRODUCTION:**

Antibiotic resistance in carbapenemase-producing *Klebsiella pneumoniae* is acquired and disseminated mainly by plasmids. Therefore, we aimed to investigate the occurrence of carbapenemase genes, analyze the genetic diversity by ERIC-PCR, and examine the most common plasmid incompatibility groups (Incs) in clinical isolates of *K. pneumoniae* from colonization and infection in patients from a hospital in Brazil.

**METHODS:**

Twenty-seven isolates of carbapenem-resistant *K. pneumoniae* were selected and screened for the presence of carbapenemase genes and Incs by PCR, followed by amplicon sequencing.

**RESULTS:**

The *bla*_KPC_ and *bla*_NDM_ genes were detected in 24 (88.8 %) and 16 (59.2 %) of the isolates, respectively. Thirteen isolates (48.1 %) were positive for both genes. The IncFIB (92.6 %) and IncQ (88.8 %) were the most frequent plasmids, followed by IncA/C, IncHI1B, and IncL/M, indicating that plasmid variability existed in these isolates. To our knowledge, this is the first report of IncHI1B in Brazil. We found eight isolates with clonal relationship distributed in different sectors of the hospital.

**CONCLUSIONS:**

The accumulation of resistance determinants, the variability of plasmid Incs, and the clonal dissemination detected in *K. pneumoniae* isolates demonstrate their potential for infection, colonization, and the dissemination of different resistance genes and plasmids.

## INTRODUCTION

*Klebsiella pneumoniae* is clinically important because it is involved in a variety of healthcare-association infections (HAI) such as in the urinary and respiratory tracts, wounds, endocarditis, and sepsis^1^ ^,^ ^2^ ^,^ ^3^. Intestinal colonization is one of the main factors favoring infection by *K. pneumoniae*, as colonized carriers can serve as important reservoirs for the spread of bacteria in the hospital environment. Further, *K. pneumoniae* can harbor resistance genes and spread them through conjugative plasmids and transposons^4^.

The excessive and indiscriminate use of beta-lactam antimicrobials has culminated in the emergence of antibiotic-resistant *K. pneumoniae* strains and other carbapenem-resistant Enterobacteria^1^ ^,^ ^5^ ^,^ ^6^ ^,^ ^7^. The development of resistance is related to the production of beta-lactamases which is mediated by conjugative plasmids^8^ ^,^ ^9^ ^,^ ^10^ ^,^ ^11^ ^,^ ^12^.

*Klebsiella pneumoniae* carbapenemase (KPC) has become endemic in several countries and is frequently detected in *K. pneumoniae* isolates from Brazilian hospitals^1^ ^,^ ^13^ ^,^ ^14^ ^,^ ^15^ ^,^ ^16^ ^,^ ^17^. The *bla*_KPC_ gene is often located on the transposon Tn4401 (Eilertson et al.^18^), which has been found in several transferable plasmids^4^ ^,^ ^19^ ^,^ ^20^, which ensures its dispersion among *Klebsiella* species and other genera of Gram-negative bacteria (Belder et al.^21^). The *bla*_KPC_ gene can also be found in non-Tn4401 elements (NTE_KPC_)^16^ ^,^ ^22^.

Additionally, resistance to carbapenems can occur due to the production of other enzymes, such as metallo-beta-lactamases, e.g., the New Delhi metallo-beta-lactamase-1 (NDM-1). Since its detection in 2008 in New Delhi, India^23^ strains producing NDM-1 have been reported in many countries, including Brazil^6^ ^,^ ^8^ ^,^ ^9^ ^,^ ^24^ ^,^ ^25^. Considering the importance of knowing which resistance genes and plasmids are circulating among multi-drug resistant clinical isolates in hospitals in Brazil, we investigated the *bla*_KPC_, *bla*_GES_, *bla*_NDM_, *bla*_VIM_, and *bla*_IMP_ genes, and the most common plasmid Incs in *K. pneumoniae* (FIB, Q, A/C, L/M, N, HI2, and HI1B) to analyze the clonal relationship between KPC resistant clinical isolates obtained from a public hospital in Recife-PE, Brazil.

## METHODS

### Bacterial isolates

Twenty-seven isolates of *K. pneumonia*e selected for being resistant to one or more carbapenems were isolated from different patients and sites of infection or colonization. The patients were admitted to a public hospital in the city of Recife-PE, Brazil, between 2017 and 2018. The isolates were kept as frozen stocks at -80ºC in 15 % glycerol.

### Antimicrobial susceptibility

The Minimum Inhibitory Concentration (MIC) for the antimicrobials Amikacin (AMI); Amoxicillin-clavulanic acid (AMC); Ampicillin (AMP); Cefazolin (CFZ); Cefepime (CPM); Cephalothin (CFL); Cefotaxime (CTX); Cefoxitin (CFO); Ceftazidime (CAZ); Ceftriaxone (CRO); Cefuroxime (CRX); Ciprofloxacin (CIP); Colistin (COL); Ertapenem (ERT); Gentamicin (GEN); Imipenem (IMI); Levofloxacin (LEV); Meropenem (MER); Piperacillin-tazobactam (PIPT); Trimethoprim-sulfamethoxazole (TRIS) was determined using automated equipment from BD Phoenix 100. The susceptibility profile was interpreted according to the guidelines provided by the Clinical and Laboratory Standards Institute (CLSI)^26^. 

### DNA extraction and PCR conditions for resistance genes

Genomic DNA was extracted using a commercial kit as per the manufacturer’s instructions (Wizard Genomic DNA Purification kit, Promega). After extraction, the DNA was quantified using the NanoDrop 2000c UV-Vis spectrophotometer. For PCR amplification of the *bla*_KPC_, *bla*_NDM_, *bla*_GES_, *bla*_VIM_, and *bla*_IMP_ genes, the primers described in Table 1 were used. The amplification reactions were prepared in a total volume of 25 μL per tube, comprising a final concentration of 25 mM MgCl_2_, 8 mM dNTPs, 1U Taq DNA Polymerase (Promega), 10 μM of each primer, 5×buffer, and 1 ng of DNA.

**TABLE 1:** Primers used in the PCR and sequencing of the amplicons in isolates of *K. pneumoniae.*

Gene	Primer	Sequence (5`- 3`)	Temp.^a^	Amplicon size (base pair)	Reference
*bla*_KPC_	KPC-1a	TGTCACTGTATCGCCGTC	63°C	800	Yigit et al. (2001)^42^
	KPC-1b	CTCAGTGCTCTACAGAAAACC			
*bla*_GES_	GES-F	GAAACCAAACGGGAGACGC	60ºC	207	Nordmann (2011)^43^
	GES-R	CTTGACCGACAGAGGCAACT			
*bla*_NDM_	NDM-F	TAAAATACCTTGAGCGGGC	52ºC	439	Nordmann (2011)^43^
	NDM-R	AAATGGAAACTGGCGACC			
*bla*_VIM_	VIM-F	CAGATTGCCGATGGTGTTTGG	62°C	600	Dong et al. (2008)^44^
	VIM-R	AGG TGGGCCATTCAGCCAGA			
*bla*_IMP_	IMP-F	GGAATAGAGTGGCTTAATTCTC	60°C	232	Dong et al. (2008)^44^
	IMP-R	GTGATGCGTCYCCAAYTTCACT			
HI-2	IncHI-2-F	GGAGCGATGGATTACTTCAGTAC	64ºC	644	Caratolli et al. (2005)^28^
	IncHI-2-R	GGCTCACTACCGTTGTCATCCT			
L/M	IncL/M-F	GGATGAAAACTATCAGCATCTGAAG	62ºC	758	Caratolli et al. (2005)^28^
	IncL/M-R	CTGCAGGGGCGATTCTTTAGG			
A/C	IncA/C-F	GAGAACCAAAGACAAAGACCTGGA	62ºC	465	Caratolli et al. (2005)^28^
	IncA/C-R	ACGACAAACCTGAATTGCCTCCTT			
N	IncN-F	GTCTAACGAGCTTACCGAAG	62ºC	559	Caratolli et al. (2005)^28^
	IncN-R	GTTTCAACTCTGCCAAGTTC			
HI1B	HI1B-Fw	CAA AAC GAG AGA TAT TCAACCC CTG ATT	63ºC	900	Caratolli et al. (2005)^28^
	HI1B-Rw	CTT GAT GAT ACA GGG			
FIB	IncFIB-F	GGAGTTCTGACACACGATTTTCTG	62ºC	702	Caratolli et al. (2005)^28^
	IncFIB-R	CTCCCGTCGCTTCAGGGCATT			
Q	Ori-V-F	CTCCCGTACTAACTGTCACG	61ºC	436	Smalla et al. (2001)^27^
	Ori-V-R	ATCGACCGAGACAGGCCCTGC			
	Rep-B-F	TCGTGGTCGCGTTCAAGGTACG	64ºC	1.160	
	Rep-B-R	CTGTAAGTCGATGATCTGGGCGTT			
	Ori-T-F	TTCGCGCTCGTTGTTCTTCGAGC	63ºC	191	
	Ori-T-R	GCCGTTAGGCCAGTTTCTCG			
NA	ERIC-1	ATGTAAGCTCCTGGGGATTAAC	36°C	NA	Duan et al., (2009)^29^
	ERIC-2	AAGTAAGTGACTGGGGTGAGCG			

**NA:** not applicable; Temp.^a^: the annealing temperature of the primers

The following thermal cycling conditions were used for *bla*_KPC_ amplification: initial denaturation for 5 min at 95ºC, followed by 30 cycles of 1 min at 95ºC for denaturation, 1 min at 63ºC for primer annealing, and 1 min at 72ºC for the extension step. Subsequently, a final elongation step of 10 min was performed at 72ºC. For amplification of the *bla*_GES_ gene, the following conditions were used: 3 min at 93°C, followed by 40 cycles of 1 min at 93°C, 1 min at 55°C and 1 min at 72°C, and a final extension for 7 min at 72°C. For the amplification of the *bla*_NDM_ gene, the conditions used were 10 min at 94°C, followed by 36 cycles of 30 s at 94°C, 40 s at 52°C, 50 s at 72°C, and a final extension of 5 min at 72°C. For the *bla*_VIM_ and *bla*_IMP_ genes, we used PCR cycling of 5 min at 95ºC, followed by 30 cycles of 1 min at 95ºC, 1 min at 60^o^C and 62^o^C for the *bla*_IMP_ and *bla*_VIM_ genes, respectively, and an extension step for 1 min at 68ºC. Subsequently, a 5-minute final elongation step was performed at 68ºC.

### Incs PCR

To detect the plasmid Incs, the primers for IncA/C, IncL/M, IncN, IncHI2, IncFIB, IncHI1B, and IncQ were selected and used^27^ ^,^ ^28^ ^,^ ^29^, as these plasmid incompatibility groups are more frequently described in the literature for *K. pneumoniae.* The cycling conditions used were 5 min at 94°C, followed by 35 cycles of 1 min denaturation at 94°C, 1 min of annealing with the specific temperature of each initiator used in the reaction (Table 1), and 1 min of extension at 72°C. The final extension was performed for 10 min at a temperature of 72°C.

### Electrophoresis and sequencing of resistance genes

The PCR products were analyzed via electrophoresis using a 1 % agarose gel in TBE buffer (0.089 M Tris-Borate and 0.002 M EDTA) at a constant voltage of 100 V. The gels were visualized under ultraviolet light using a transilluminator (Bio Rad) and photographed with a photo-documentation system (Photocap, Vilber Lourmat). The amplicons for each gene were purified using the SV Total DNA Isolation System (Promega) and the DNA was sequenced by the method of Sanger et al. (1997). The nucleotide sequences were analyzed using the BLAST program (http://www.ncbi.nlm.nih.gov/).

Enterobacterial Repetitive Intergenic Consensus Polymerase Chain Reaction (ERIC-PCR)

To assess the clonal relationship between the isolates, ERIC-PCR was performed as described by Duan et al. 2009^29^ and Cabral et al. 2012^14^ (Table 1). The DARWIN 6.0 software was used to generate the dendrogram.

## RESULTS

### Antimicrobial resistance profile

Although isolates resistant to at least one carbapenem were selected, we observed that most isolates were resistant to three carbapenems tested (i.e., ertapenem, imipenem and meropenem), except for isolate K5-A3 which was sensitive to imipenem and showed intermediate sensitivity to meropenem, and the K6-A3 isolate which had intermediate sensitivity to imipenem (Table 2). The antimicrobials that showed the best activity against carbapenem-resistant *K. pneumoniae* isolates were amikacin and colistin, showing that 96.2% and 88.9% were sensitive to these antimicrobials, respectively.

**TABLE 2:** The source of isolation; minimum inhibitory concentration (MIC) for ertapenem (ERT), imipenem (IMI), and meropenem (MER); genes for carbapenemases (Resistance genes); plasmid Incs (Incs); and the ERIC-PCR profile of *K. pneumoniae* isolates from a public hospital in Recife-PE, Brazil.

Isolates	Sector	Clinical sample	MIC(ERT)	MIC(IMI)	MIC(MER)	Resistance Genes	Incs	ERIC-PCR
K5-A3	ICU	Surgical drain	>4(R)	<=1(S)	2(I)	*bla*_KPC_	Q, FIB	E7
K6-A3	CU	Blood	>4(R)	2(I)	4(R)	*bla*_KPC_	Q, FIB	E2
K8-A3	ICU	Urine	>4(R)	>8(R)	>8(R)	*bla*_KPC,_ *bla*_NDM_	Q, FIB	E2
K9-A3	GE	Blood	>4(R)	>8(R)	>8(R)	*bla*_KPC,_ *bla*_NDM_	Q, FIB	E7
K10-A3	ICU	Blood	>4(R)	>8(R)	>8(R)	*bla*_KPC_	Q, FIB, HI1B	E4
K11-A3	CU1	Blood	>4(R)	8(R)	4(R)	*bla*_KPC_	Q, HI1B	E12
K12-A3	ICU	Blood	>4(R)	>8(R)	>8(R)	*bla*_KPC,_ *bla*_NDM_	Q, FIB	E1
K16-A3	Cardiology	Urine	>4(R)	>8(R)	>8(R)	*bla*_NDM_	FIB	E3
K24-A3	ICU	Urine	>4(R)	>8(R)	>8(R)	*bla*_KPC,_ *bla*_NDM_	Q, FIB, A/C	E1
K26-A3	GE	Urine	>4(R)	8(R)	4(R)	*bla*_KPC,_ *bla*_NDM_	Q, FIB	E11
K27-A3	MC	Urine	>4(R)	>8(R)	>8(R)	*bla*_KPC,_ *bla*_NDM_	Q, FIB	E1
K30-A3	CU1	Blood	>4(R)	>8(R)	>8(R)	*bla*_KPC,_ *bla*_NDM_	Q, FIB	E1
K31-A3	ICU	Urine	>4(R)	>8(R)	>8(R)	*bla*_KPC_	FIB, A/C	E1a
K2-A3	Cardiology	Rectal Swab	>4(R)	(R)	(R)	*bla*_KPC_	Q, FIB, L/M	E5
K3-A3	CU2	Rectal Swab	>4(R)	>8(R)	>8(R)	*bla*_KPC,_ *bla*_NDM_	Q, FIB	E7
K13-A3	CU1	Rectal Swab	>4(R)	>8(R)	>8(R)	*bla*_KPC_	Q, FIB, A/C	E10
K14-A3	CU2	Rectal Swab	>1(R)	>8(R)	>32(R)	*bla*_KPC_	Q, FIB, HI1B	E4
K15-A3	Cardiology	Rectal Swab	>1(R)	>8(R)	>32(R)	*bla*_KPC_	Q, FIB	E6
K17-A3	ICU	Rectal Swab	>4(R)	>8(R)	>8(R)	*bla*_KPC,_ *bla*_NDM_	Q	E2
K18-A3	CU1	Rectal Swab	>4(R)	>8(R)	>8(R)	*bla*_KPC_	Q, FIB	E1
K20-A3	CU1	Rectal Swab	>4(R)	>8(R)	>8(R)	*bla*_NDM_	FIB	E1
K21-A3	Cardiology	Rectal Swab	>4(R)	>8(R)	>8(R)	*bla*_KPC_	Q, FIB, A/C	E1a
K29-A3	CU	Rectal Swab	>4(R)	>8(R)	>8(R)	*bla*_KPC,_ *bla*_NDM_	Q, FIB	E1
K32-A3	ICU	Rectal Swab	>4(R)	>8(R)	>8(R)	*bla*_KPC,_ *bla*_NDM_	Q, FIB	E8
K34-A3	ICU	Rectal Swab	>4(R)	>8(R)	>8(R)	*bla*_NDM_	Q, FIB	E1a
K36-A3	CU2	Rectal Swab	>4(R)	>8(R)	>8(R)	*bla*_KPC,_ *bla*_NDM_	Q, FIB, L/M	E9
K37-A3	ICU	Rectal Swab	>4(R)	>8(R)	>8(R)	*bla*_KPC,_ *bla*_NDM_	Q, FIB	E1

K: *Klebsiella pneumoniae*; **GE:** General Emergency; **MC:** Medical Clinic; **ICU:** Intensive care unit; **CU:** Coronary Unit; **MIC:** Minimal Inhibitory Concentration; **ERT:** Ertapenem, **IMI:** Imipenem, **MER:** Meropenem, **R:** Resistant; **I:** Intermediate; **S:** Sensitive; **A3:** public hospital; +, presence of the gene; - absence of the gene; Shaded text: clinical isolates from colonization.

### Beta-lactamase genes

The *bla*_KPC_ and *bla*_NDM_ genes were detected in 24 (88.8 %) and 16 (59.2 %) isolates of *K. pneumoniae,* respectively (Table 2), by amplifying the expected 800 bp and 621 bp genes for *bla*_KPC_ and *bla*_NDM_ respectively. The *bla*_GES_, *bla*_VIM,_ and *bla*_IMP_ genes were not detected. The *bla*_KPC_ and *bla*_NDM_ genes were detected in 13 (48.1 %) of the isolates analyzed. The *bla*_KPC-2_ and *bla*_NDM-1_ variants were confirmed by sequencing the PCR product from representative isolates.

### Plasmid Incompatibility Groups (Incs)

The FIB (n=25; 92.6 %) and Q (n=24; 88.8 %) Incs were the most frequent in the *K. pneumoniae* isolates analyzed in this study, followed by the Incs A/C, HI1B, and L/M that were detected in 4 (14.8 %), 3 (11.1 %) and 2 (7.4 %) isolates, respectively. The Incs N and HI2 were not detected. Two isolates (K16-A3 and K20-A3) showed the presence of the IncFIB alone, and the isolate K17-A3 had only IncQ. The remaining 24 isolates had more than one Inc that were investigated in this study (Table 2).

### Molecular typing by ERIC-PCR

**FIGURE 1:**
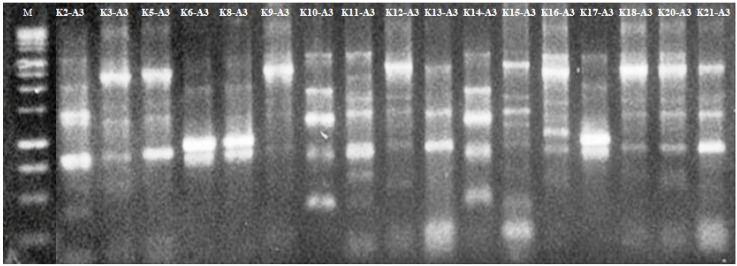
Electrophoresis using a 1.5 % agarose gel for ERIC-PCR from representative isolates of *K. pneumoniae*. Lane 1: 1 Kb molecular weight marker (Promega), lanes 2-17: *K. pneumoniae* isolates.

**FIGURE 2:**
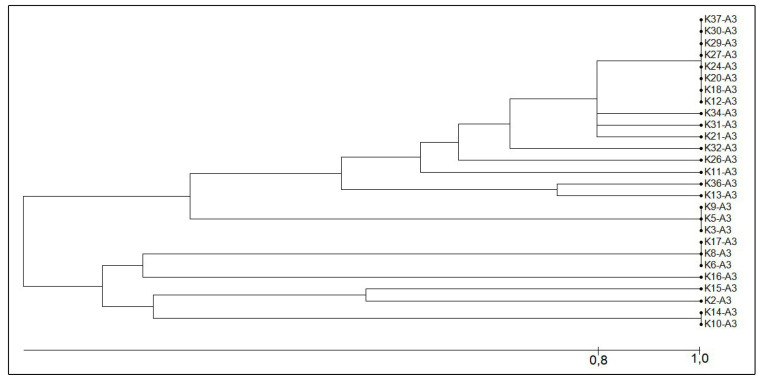
Dendrogram generated from the results of the ERIC-PCR using the Darwin 6.0 software, illustrating the relationship between the profiles of the 27 isolates of *K. pneumoniae* that were resistant to carbapenems and obtained from Recife-PE, Brazil.

Of the 27 isolates of *K. pneumoniae* analyzed, 12 different genetic profiles were identified (Figure 1 and Figure 2) by ERIC-PCR. Eleven isolates showed an 80 % similarity (E1a profile), of which eight isolates showed a 100 % similarity (E1 profile). Four isolates showed an identical resistance profile (presence of the *bla*_KPC_ and *bla*_NDM_ genes) and the presence of the same plasmid Incs (Q and FIB). The E2 profile grouped three isolates that were different for their resistance profile and the presence of Incs. The E4 profile grouped two isolates that showed an identical resistance profile (only the *bla*_KPC_ gene was detected) and Incs (Q, FIB and HI1B). The E7 profile grouped three isolates, of which two isolates were identical for their resistance profile (*bla*_KPC_ and *bla*_NDM_ gene) and in the presence of Incs (Q and FIB), whereas one isolate in this profile had the *bla*_KPC_ gene alone and the same plasmid Incs. The isolates that were not clonally related had a different resistance profile and the presence of distinct plasmid Incs.

Comparative analysis of the presence of genes for carbapenemases, plasmid Incs, and ERIC-PCR, based on colonization or infection as sources for isolation.

Of the 27 carbapenem-resistant *K. pneumoniae* isolates, 12 were from infected samples (blood or urine), 14 were from colonization samples (rectal swab), and 1 was from a cavity drain (Table 2). The majority (58.3 %) of the isolates from the infected samples that had the *bla*_NDM_ gene also harbored the *bla*_KPC_ gene, except for the K16-A3 isolate that harbored the *bla*_NDM_ gene alone. On analyzing the isolates from colonization samples, we observed that six isolates were positive for the *bla*_KPC_ and *bla*_NDM_ genes. The Incs FIB, Q, A/C, and HI1B were detected in isolates from infection and colonization, and IncL/M was detected in only two isolates from colonization.

## DISCUSSION

Most of the *K. pneumoniae* isolates evaluated in this study were resistant to all beta-lactams tested. These data justify the alert published by the CDC in 2013, which states that carbapenemase-producing enterobacteria are a global threat due to the high rates of resistance to antimicrobials, and urgent and effective actions are required to control them^30^. Lorenzoni et al. (2017)^31^ performed studies with carbapenem-resistant enterobacteria isolated from a hospital in the Rio Grande do Sul, Brazil, and detected high rates of sensitivity to colistin and amikacin in *K. pneumoniae* isolates. Further, the authors found that the *bla*_KPC_ gene was detected in 80 % of the isolates, corroborating the data in this study, where we found an occurrence of 88.8 % for the *bla*_KPC_ gene. These occurrence rates of the *bla*_KPC_ gene identified in this study highlight the persistence of this gene in *K. pneumoniae*, and since its detection in 2006 from Recife, Brazil^13^, it remains the main carbapenemase associated with carbapenem-resistant *K. pneumoniae* samples in several Brazilian states, including Recife-PE^1^ ^,^ ^5^ ^,^ ^32^.

The occurrence of the *bla*_NDM_ gene in 59.2 % of the isolates analyzed in this study is worrying and highlights the emergence of another carbapenemase that, in addition to KPC, can also hydrolyze carbapenems. Barberino et al. (2018)^33^ were the first to report the presence of the *bla*_NDM_ gene in clinical isolates of *K. pneumoniae* and *Citrobacter* in two patients admitted to a public hospital in Salvador, Bahia, in northeastern Brazil. Da Silva et al. (2019)^34^ detected the *bla*_NDM-1_ gene in different species of Gram-negative bacteria isolated from nine Brazilian states but did not include the state of Pernambuco. Scavuzzi et al. (2019)^35^ detected an isolate of *K. pneumoniae* that in addition to harboring the *bla*_NDM_ gene, also harbored bacterial virulence genes, and were the first to report strains carrying the *bla*_NDM_ gene in bacterial isolates from Recife-PE. Additionally, Firmo et al. (2019)^17^ detected the occurrence of *bla*_NDM_ in 25 % of *K. pneumoniae* isolates, and also in isolates from Recife-PE. In our study, we detected a greater number of isolates with the *bla*_NDM_ gene (n=16; 59.2 %), and these results indicated the rapid dissemination of this gene.

The rate of occurrence of the *bla*_NDM_ and the *bla*_KPC_ genes deserves to be highlighted because of the accumulation of these genetic mechanisms of resistance in the same bacterial species. In Brazil, the accumulation of resistance determinants in *K. pneumoniae* has been described by other authors. Nava et al. (2018)^7^ detected the occurrence of the *bla*_NDM_ *, bla*_KPC_, and *bla*_TEM_ genes in clinical isolates of *K. pneumoniae* from a university hospital in Londrina-PR. The concomitant presence of the *bla*_KPC_ and *bla*_NDM_ genes in colonization isolates examined in this study is worrying and reinforces the need for surveillance cultures, as patients showing bacterial colonization are an important reservoir for the spread of resistance mechanisms within the hospital environment and are the main gateway to the development of infection.

We observed that *K. pneumoniae* isolates, despite being clonally related as indicated by an ERIC-PCR assay, presented different types of plasmids and different resistance genes. This may occur because the ERIC-PCR technique amplifies repetitive intergenic regions of the bacterial chromosome, yet does not necessarily amplify plasmid regions, where most of the resistance genes are located^36^. Clonal dissemination of the isolates was observed in different sectors of the hospital under study and among colonized and infected patients. Therefore, our results indicate that *K. pneumoniae* can potentially spread in the hospital environment.

The persistence of genes that confer resistance to carbapenems results due to the clonal dissemination of the isolates and via the dispersion of these genes through conjugative or mobile plasmids^8^ ^,^ ^9^ ^,^ ^10^ ^,^ ^11^. In this study, we detected five types of Incs which are described in the literature as being potentially responsible for the spread of *bla*_KPC_ and *bla*_NDM_ genes in *K. pneumoniae* isolates. Additionally, all Incs identified in this study already harbor resistance genes, including *bla*_KPC_ and *bla*_NDM_ ^4^ ^,^ ^16^ ^,^ ^19^ ^,^ ^20^ ^,^ ^37^ ^,^ ^38^, and the Incs FIB and Q were the most frequently detected in this study. This study is the first report of the IncFIB in bacterial isolates from Recife-PE, Brazil. IncFIB is a conjugative plasmid that has been associated with the dissemination of the *bla*_IMP_ gene in *E. cloacae* in Japan^39^, and was also reported in Africa in *E. coli* isolates carrying the *bla*_TEM_ gene^40^. In Europe, it was responsible for the spread of the *bla*_NDM-1_, *bla*_SHV-12_, *bla*_CTXM-15_, and *bla*_OXA-1_ genes in *K. pneumoniae* ^11^.

The second most frequently detected Inc in *K. pneumoniae* isolates was IncQ, a plasmid that harbors carbapenem resistance genes and had been gaining prominence in some regions of Brazil. Nicoletti et al. (2015)^41^ identified the *bla*_KPC_ gene inserted into an IncQ plasmid in *K. pneumoniae.* Cerdeira et al. (2019)^16^ detected two isolates of *K. pneumoniae* that were resistant to carbapenems and had the *bla*_KPC_ gene inserted into IncQ plasmids. According to Smalla et al. (2000)^27^, IncQ is a small plasmid that can vary between 5.1-14.2 kb in size, and it can be found in several host bacterial cells. IncQ is not conjugative, but is mobilizable and promiscuous, and can be transferred from one bacterium to another by conjugative plasmids, which are present in the same bacterial cell. Given the variability in plasmids, including for conjugative plasmids, which were detected in this study in *K. pneumoniae* isolates, IncQ can probably be disseminated to other species. Additionally, it plays an important role in enhancing the dissemination of the *bla*_KPC_ gene in Brazil^16^.

Incs A/ C and L/M, despite being detected in a smaller number of isolates in this study, have been described in previous studies as carrying resistance genes in different species of enterobacteria in Brazil, including *K. pneumoniae* ^20^. Pereira et al. (2015)^37^ detected the simultaneous presence of the *bla*_KPC_ and *bla*_NDM_ genes in *E. hormaechei* in Rio de Janeiro, with *bla*_NDM_ inserted into IncA/C. Only one study has investigated Incs in *K. pneumoniae* isolates from Recife-PE, Brazil; however, the plasmids were not typed, and the small number of isolates analyzed (only four) may be a limitation of the study^19^.

We also detected the presence of the conjugative plasmid IncHI1B in this study, and to our knowledge, this is the first report of this Inc in Brazil. This plasmid has been reported in clinical isolates of *E. cloacae, K. pneumoniae, E. coli*, and *C. freundii* as carrying the *bla*_NDM_ gene in hospitals in the United States of America^42^. Additionally, Al Baloushi et al. (2018)^10^ detected isolates of *K. pneumoniae* carrying the *bla*_NDM_ gene in IncHI1B in Saudi Arabia. Matsumura et al. (2018)^38^ performed a conjugation and transformation experiment in isolates from surveillance programs and identified the *bla*_VIM_ gene in plasmids IncL/M, IncN2, IncHI1B, and IncFIB in *K. pneumoniae* isolates from Greece and Spain. These studies show the ability of IncHI1B to host genes for carbapenemases in different species.

We conclude that the accumulation of resistance determinants, the variability of plasmid Incs, and the clonal dissemination of these in *K. pneumoniae* isolates from infection and colonization, demonstrate the ability of this species to acquire genes for resistance and disseminate them via conjugative and mobilizable plasmids. The importance of the early phenotypic and genetic identification of resistance mechanisms in bacterial isolates from infection and colonization samples needs to be highlighted to prevent and halt the development of infection in patients hospitalized due to immuno-depression.
